# The first 60 cases of robotic sacrocolpopexy with the novel HUGO RAS system: feasibility, setting and perioperative outcomes

**DOI:** 10.3389/fsurg.2023.1181824

**Published:** 2023-05-05

**Authors:** Giovanni Panico, Lorenzo Vacca, Giuseppe Campagna, Daniela Caramazza, Sara Mastrovito, Andrea Lombisani, Alfredo Ercoli, Giovanni Scambia

**Affiliations:** ^1^ Dipartimento di Scienze Della Salute Della Donna e del Bambino e di Sanità Pubblica, Università Cattolica del Sacro Cuore, Fondazione Policlinico Universitario A. Gemelli IRCCS, UOC Chirurgia Ginecologica, Roma, Italy; ^2^ PID Ginecologia Oncologica e Chirurgia Ginecologica Miniinvasiva, Università Degli Studi di Messina, Policlinico G. Martino, Messina, Italy

**Keywords:** HUGO RAS, robotic sacrocolpopexy, pelvic organ prolapse, pelvic reconstructive surgery, robotic surgery

## Abstract

**Introduction:**

We present the preliminary report of the first 60 cases of robotic sacrocolpopexy (RSCP) performed with a minimally invasive approach by using the new HUGO RAS system (Medtronic) with the aim of assessing its feasibility, safety and efficacy.

**Methods:**

Results in terms of operative time, intraoperative blood loss, post-operative pain, length of hospitalisation, intra and post-operative complications were comparable to previously described laparoscopic and robotic techniques.

**Results:**

Urogynecological assessment at three months follow up showed surgical anatomic success in 96.7% of patients (<2 POP-Q stage), while subjective cure rate was 98.3%.

**Conclusions:**

This is the first series analyzing RSCP outcomes for POP using the new Hugo RAS system. Our results suggest effectiveness both in objective and subjective outcomes, with minimal intra and post-operative complications. Larger series as well as longer follow-up are needed to better define advantages and possible disadvantages of this novel system. Our work may represent the basis of future studies to confirm its safety, efficacy and feasibility, and may provide technical notes for other centres that wish to perform RSCP through this innovative system.

## Introduction

Pelvic organ prolapse (POP) is a common condition negatively affecting quality of life of a consistent percentage of women. Lifetime risk of a woman undergoing POP-related surgery is estimated to be 11% (1). Minimally invasive sacrocolpopexy is nowadays considered the gold standard for the surgical treatment of apical and multicompartmental prolapse, combining high success rates with low risk of recurrence compared to other techniques (2). Over the last decade the goal of new surgical innovations in minimally invasive surgery (MIS) has been to reduce complications related to open abdominal surgeries, preserving its efficacy and safety.

Robot-assisted surgery (RAS) is nowadays well established for major surgery across the world, and its indications include the management of many benign and malignant diseases in urology and gynecology. The introduction of the first robotic system (da Vinci, Intuitive Surgical System) determined improvements in terms of surgical learning curve and feasibility of MIS. The da Vinci emerged as the predominant system in RAS and is up to today the most widely available platform. In recent years, other robotic platforms have emerged, including Versius by Cambridge Medical Robotics; ALF-X by Senhance; Revo-i by Meere, Micro Hand S by Tianjin University.

In this scenario of rapid technological evolution supported by robust evidence of the advantages of MIS over laparotomy, attention has now turned to whether there are additional benefits conveyed by these newly introduced robotic systems. In particular, there is a need to define their potential advantages as well as their limits in comparison to pre-existing techniques and systems.

One of the newest systems on the market is Hugo RAS (MEDTRONIC Inc, USA). The new robotic platform consists of a remote open surgical console, independent manipulator arms and a connection node. Some of its features include remote HD–3D display with an eye-tracking camera control system, integrated haptic interaction, and high configuration versatility. A Karl Storz 3D Tipcam STM (Karl Storz SE & Co. KG, Tuttlingen, Germany) encased in a robotic adaptor provides endoscopic vision. Independent arms offer multiple degrees of freedom, that should exponentially increase the range of movement, allowing the carts to be kept at a distance from the patient, at the expense of needing more space in the operatory room.

Accepting a new surgical robot in clinical practice mandates the demonstration of technical feasibility, efficacy and clinical safety. Hugo RAS has been introduced in Europe in March 2022, receiving CE (Conformité Européenne) approval for gynaecological and urological procedures. In the United States, the system has not yet received FDA approval. In the last few months, some preliminary reports in urologic and gynecologic surgery have been published (3–6).

As some of the earliest adopters of this technology (7), we report our initial experience of robotic sacrocolpopexy (RSCP), using the new HUGO RAS system.

The aim of this report was to assess the feasibility and provide technical details of the setup for sacrocolpopexy, on our large case series. In addition, our preliminary insights on this new platform may be interesting to other centers that may soon introduce this new robotic system.

## Materials and methods

We hereby present the results regarding the first 60 patients that underwent RSCP using the new Hugo RAS system at the Urogynecology Division of the Gynecology department of Fondazione Policlinico Universitario A. Gemelli IRCCS.

Here follow preliminary results and patient baseline data. All patients were affected by multicompartmental prolapse. Before surgical procedure, patients went through clinical examination and instrumental preoperative workup including detailed medical history, physical examination, POP-Q scores evaluation, laboratory exams, pelvic and urinary tract ultrasound, PAP test and a urodynamic examination.

All patients received a prior detailed description of the procedure by signing an informed consent.

During surgery, intraoperative data as well as specific time parameters were measured:

 • Docking time was defined as the time to change and adapt the robotic setting to the patient.
 • Operative time was defined as the interval from the start of procedure to the suture of surgical incisions.
 • Console time was considered from the moment the first operator started the procedure at the robotic console, until the end of its usage.
 • Intraoperative complications were defined as any bowel, bladder, ureteral, or vascular injury, estimated blood loss (EBL) exceeding 500 ml, need for intra- or post-operative blood cell transfusion or any other unplanned event.
 • Post-operative complications were analyzed according to the Clavien-Dindo classification, defining them defined as any adverse events that occurred within 30 days from surgery.
 • Post-operative pain was assessed using a validated visual analog pain scale (VAS) and was subjectively reported at 2, 4, 12, and 24 h after surgery.
 • Duration of the hospital stay was calculated from the day after surgery (day 1) to discharge.

Two senior surgeons, with the experience of more than 50 minimally invasive sacrocolpopexy per year were selected for the present study.

**Figure 1:**
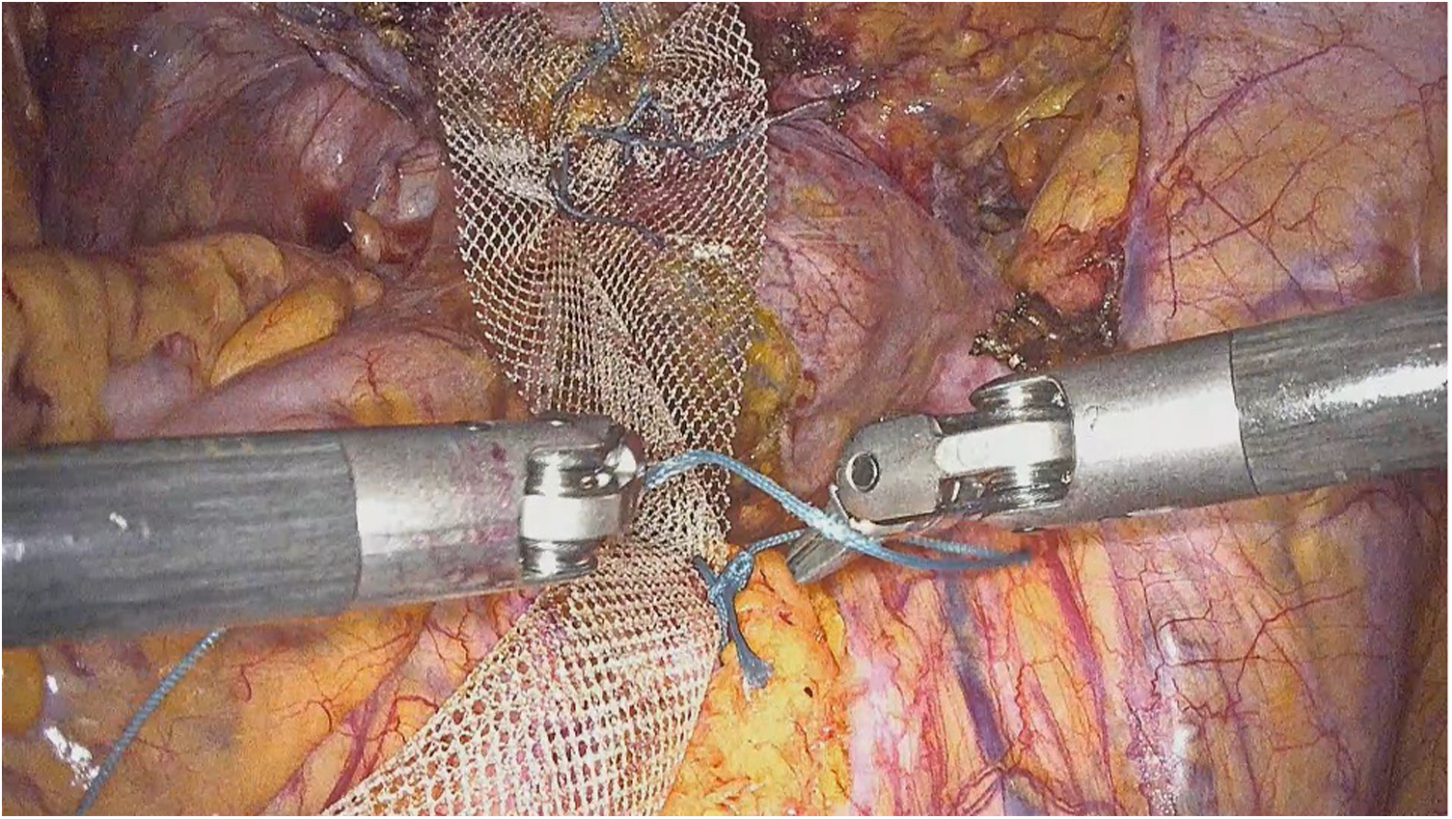
Intraoperative view during the mesh suspension to the sacrum.

**Figure 2:**
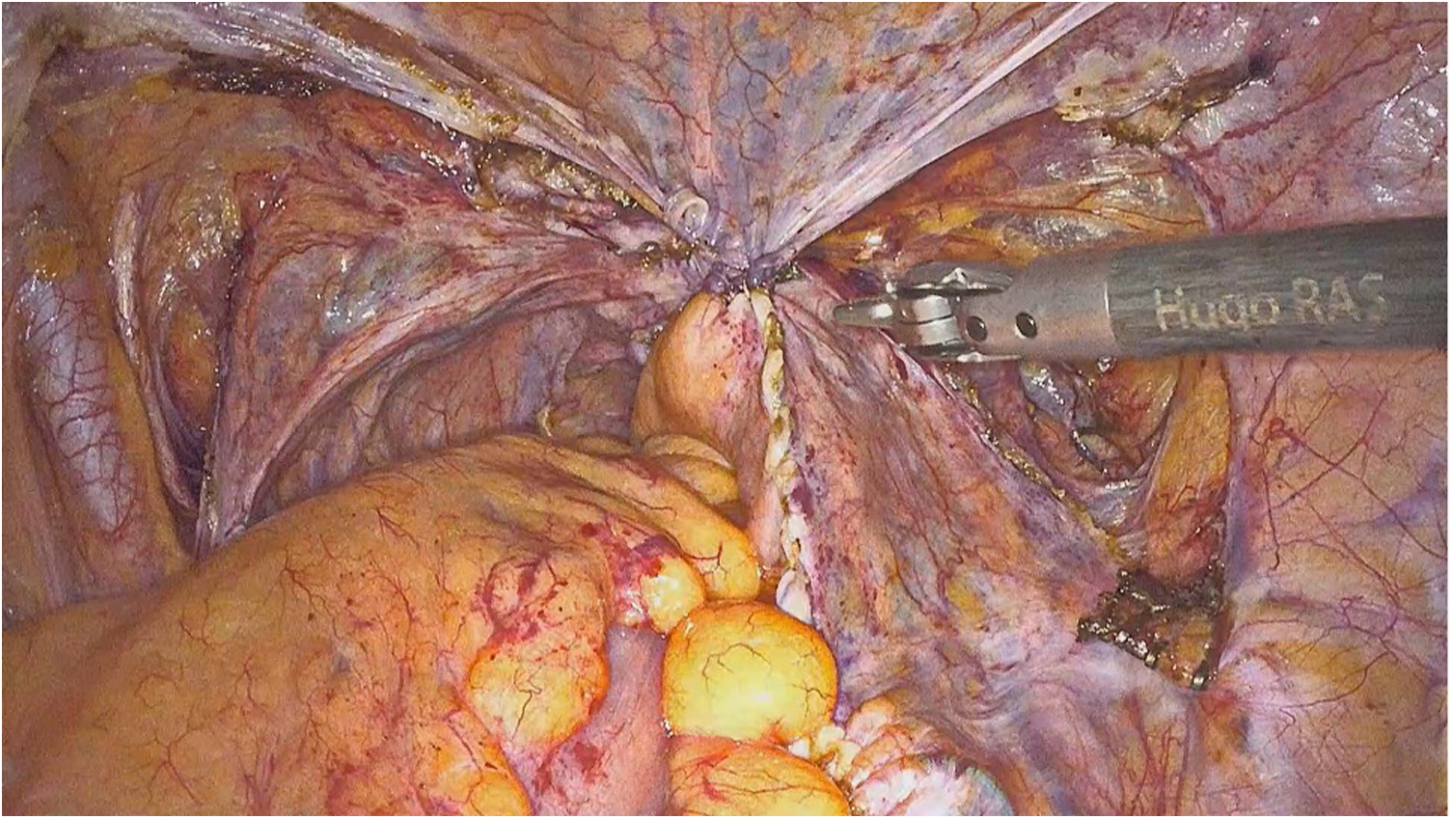
Intraoperative view at the end of the procedure.

The surgical procedure was carried out by a standardized technique as previously described by our group (8, 9) (Figures 1, 2). In post-menopausal non-hysterectomized patients that desired it, the procedure also consisted of a subtotal hysterectomy as well as a salpingo-oophorectomy.

Categorical data are presented as the number of patients and a percentage. The median and range were used for skewed data. The SPSS statistical software program (SPSS Inc., Chicago, IL) was used.

During the months from April 2022 to July 2022 sixty patients were enrolled in this study and underwent RSCP using the Hugo RAS system. The baseline characteristics of the study population are summarized in Table 1. Pelvic Organ Prolapse evaluation was described in accordance with the ICS/IUGA joint report on the terminology for pelvic floor dysfunction.

**Table 1:** Baseline characteristics.

All cases	60
Age (year), median (range)	66 (44–84)
BMI (kg/m^2^), median (range)	24.25 (20.31–31.63)
Parity (*n*), median (range)	2 (0–5)
Menopausal, *N* (%)	56 (93.3%)
Previous abdominal surgery, *N* (%)	35 (58.3%)
Previous POP surgery, *N* (%)	5 (13.3%)
Previous hysterectomy, *N* (%)	10 (16.7%)
Preoperative POP-Q stage, *N* (%)	
3	28 (46.7%)
4	32 (53.3%)
Anterior POP-Q stage, median (range)	3 (1–4)
Apical POP-Q stage, median (range)	3 (2–4)
Posterior POP-Q stage, median (range)	1 (0–4)

### Trocar positioning and docking

After general anaesthesia, patients were positioned in a lithotomy position. The procedure started by inserting a 12 mm optic port in umbilical position. Once pneumoperitoneum at 12 mmHg was reached, a 3D-HD 0° 10 mm scope (Karl Storz Endoscopy) was inserted. Two additional 8 mm ports were placed under direct visualization in the right and left lower abdomen at 11 cm–13 cm distance from the umbilical port and 5 cm below the transumbilical plane. For those two trocars, a minimal distance of 8 cm is required to avoid collisions between arms. An additional 5 mm trocar was placed at palmer's point, for the first assistant's use.

Each arm requires its own settings, that may be adjusted depending on the patient's body type. Two main settings are required to configure each arm. The first is the tilt angle, which is the vertical angle between the arm and the operative field which can be adjusted by lifting upwards or downwards the arm's nose. The second setting is the docking angle, which is the clockwise horizontal angle between the patient's head and the arm's direction. A three robotic arms configuration was chosen in all cases, following the “compact” “bridge” configuration, already described by Gueli Alletti et al. (4). In particular, the docking specifics are listed in Table 2.

**Table 2:** Docking specifics.

Arm 1	Left hand	Tilt angle +30°	Docking angle 100°
Arm 2	Optic port	Tilt angle −45°	Docking angle 150°
Arm 3	Right hand	Tilt angle −45°	Docking angle 220°

Small adjustments were made during docking to optimize the angles necessary for each patient. In all procedures, for the dissection part a bipolar grasper was used for the left surgeon's hand and a monopolar curved scissors for the right hand. During the reconstructive part of the procedure two needle holders were used by surgeons to fix the mesh. Different graspers, metallic clip applicator and suction irrigation cannula were used by the first assistant surgeon through the 5 mm port.

The surgical procedure resembled that of laparoscopic sacrocolpopexy and has been previously described extensively by our group (9–12).

## Results

All patients had POP-Q stage 3–4 multicompartmental prolapse. Ten patients (16.7%) had previously undergone a total hysterectomy as well as a salpingo-oophorectomy. Thirty-five patients (58.3%) had previous laparotomic abdominal surgery, 8 patient (10%) had previous POP repair surgery.

Perioperative data is reported in Table 3. The median operative time (OT) was 185 min (range 95–305). The median docking time was 4 min (range 2–12). Median estimated blood loss was 15 ml (range 10–100). For all procedures performed, no conversion to laparotomy was recorded. Four patients had adhesions requiring laparoscopic adhesiolysis before robotic docking. Fifty-six patients had associated procedures, including 49 subtotal hysterectomies, 1 total hysterectomy, 49 salpingo-oophorectomies. Four patients had combined nerve-sparing sacrocolpopexy and ventral rectopexy for associated rectal prolapse and obstructed defecation syndrome.

**Table 3:** Peri-operative data.

Variables	
No. of patients	60
Associated surgical procedures, *N* (%)	56 (93.3)
Ventral rectopexy, *N* (%)	4 (6.7)
Subtotal hysterectomy, *N* (%)	49 (81.7)
Total hysterectomy, *N* (%)	1 (1.7)
Salpingo-oophorectomy, *N* (%)	49 (81.7)
Others, *N* (%)	6 (10)
Docking time (min), median (range)	4 (2–12)
Console time (min), median (range)	134 (49–235)
Operative time (min), median (range)	185 (95–305)
Laparoscopic adhesiolysis, *N* (%)	4 (6.7)
EBL (ml), median (range)	15 (10–100)
Time to discharge (days), median (range)	3 (2–4)
Conversion, *N* (%)	
LPS	0
LPT	0
Intraoperative compilcations, *N* (%)	1 (1.7)
Post-operative complications, *N* (%)	4 (6.7)
Grade 1	1 (1.7)
Grade 2	3 (5)
VAS score, median (range)	
2 h	2 (1–3)
4 h	2 (1–3)
12 h	4 (1–8)
24 h	3 (1–5)

One minor intraoperative complication was reported, specifically a small opening of the anterior vaginal wall repaired intraoperatively with no post-operative consequences. Four 30-day post-operative complications were registered. Specifically, 1 mild fever treated with oral analgesics, 2 lower urinary tract infections treated with oral antibiotics, 1 umbilical infection treated with oral antibiotics. Median time to discharge was 3 days (2–4). Pain VAS score decreased after surgery, with a 24 h median of 3 (range 1–4).

Median follow up was 4-months (3–6). No case of mesh extrusion was reported. Out of the 23 patients that complained stress urinary incontinence preoperatively, 10 did not complain about the symptom after surgery (43.5%). Two *de novo* stress urinary incontinence were reported, whilst no patient developed post-operative *de novo* urge incontinence.

Anatomical and functional outcomes are reported in Table 4. Follow-up at three months demonstrated statistically significant improvement of outcome parameters using the POP-Q classification. Anatomical cure rate was 96.7% with only two cases of anatomical recurrences of the anterior compartment (POP-Q stage 2 and 3 respectively). We registered a statistically significant bulge symptom resolution in all patients. Subjective cure rate was 98.3%. No significant differences regarding pre- and post-operative constipation symptoms were observed, and no other *de novo* symptoms were registered. Patient-reported outcome data at 3 months showed that all 59 (98.3%) women had PGI-I scores 1–2.

**Table 4:** Anatomical and functional outcomes.

POP-Q stage, median (range)	Preoperative	Post-operative	*P* value
Anterior	3 (1–4)	0 (0–3)	**0** **.** **001**
Apical	3 (2–4)	0 (0–1)	**0**.**001**
Posterior	1 (0–4)	0 (0–1)	**0.001**
Stress urinary incontinence, *N* (%)	23 (38.3)	15 (25)	0.248
Urgency	29 (48.3)	10 (16.7)	**0**.**001**
Nicturia	11 (18.3)	5 (8.3)	0.210
Urge urinary incontinence	16 (26.7)	5 (8.3)	**0.001**
Hesitancy	44 (73.3)	3 (5)	**0**.**001**
Feeling of incomplete emptying	38 (63.3)	3 (5)	**0.001**
Constipation	19 (31.7)	13 (21.7)	0.238
Vaginal bulging	60 (100)	1 (1.7)	**0.000**
PGI-I, median (range)		1 (1–3)	

Bold value means Statistically significant results.

## Discussion

Robotic surgery gained popularity over the last decades, due to its possible advantages for both surgeons and patients. Among those, increased accuracy, faster suturing, reduced number of surgical errors, multiaxial movements, lack of hand tremor and surgeon comfort (13).

A systematic review published in 2014 by Serati et al. analyzed RSCP outcomes of 27 studies from 2006 to 2013 with a total of 1,488 patients, showing various objective and subjective outcomes, as well as perioperative outcomes (14). They found how conversion rate to open surgery was <1% (range: 0%–5%); intraoperative, severe post-operative complications, and mesh erosion rates were 3% (range: 0%–19%), 2% (range: 0%–8%), and 2% (range: 0%–8%), respectively. Median operative time was 194 min (75–537), estimated blood loss of 50 ml (10–1,000), and hospital stay of two days (0–50), which were all similar to our findings.

Operative times varied among different studies reflecting the execution of concomitant procedures, (hysterectomies and anti-incontinence procedures in 38% and 33% of cases, respectively). Given that our proportion of concomitant hysterectomy was higher (93.3% of concomitant surgeries, including hysterectomy in 83.4% of cases, ventral rectopexy in 6.7% of cases, and non-gynecologycal abdominal procedures in 10% of patients), our results in term of median operative time of 185 min (range 95–305) seem to be promising. In fact, considering the effective median console time of 134 min (range 49–235), it is likely that in our series the procedure of RSCP was performed more efficiently than in most of the studies reported in the systematic review. This may be related to the high experience of the surgeons that performed the procedures in this series, as well as the high-volume centre.

In terms of objective and subjective outcomes, Serati et al. reported a range from 84% to 100% and from 92% to 95%, respectively (14). In this series, we report an anatomical cure rate of 96.7% with only two cases of anterior recurrence (POP-Q stage 2 and 3 respectively) and no cases of apical recurrence. Subjective cure rate of 98.3% was registered through the use of PGI-I scores and validated questionnaires. Thus, our findings seem to be consistent with what previously published in literature, although a longer follow up of our patients is certainly needed to have a higher accuracy.

As we previously said, robotic surgical procedures were introduced to overcome some technical difficulties and the steep learning curve of laparoscopic surgery. Features such as increased magnification, three-dimensional vision, stereotaxis, physiologic tremor filtering, and a more intuitive control of the surgical instruments allow surgeons to perform complex procedures such as SCP in a comfortable setting simplifying complex tasks of vaginal dissection and laparoscopic suturing.

A recent meta-analysis by Chang et al. compared laparoscopic and robotic gynecologic procedures, finding a lower intraoperative blood loss and shorter hospital stay in the robotic group, with little or no difference in complication rates (15). Our findings demonstrated that surgery did not differ from laparoscopic procedure in terms of intra and post-operative complications, hospital stay, and blood loss (12). The only differing aspect was OT, that was inevitably longer compared to standard laparoscopy, but similar to timings described for RSCP (14, 15).

**Figure 3:**
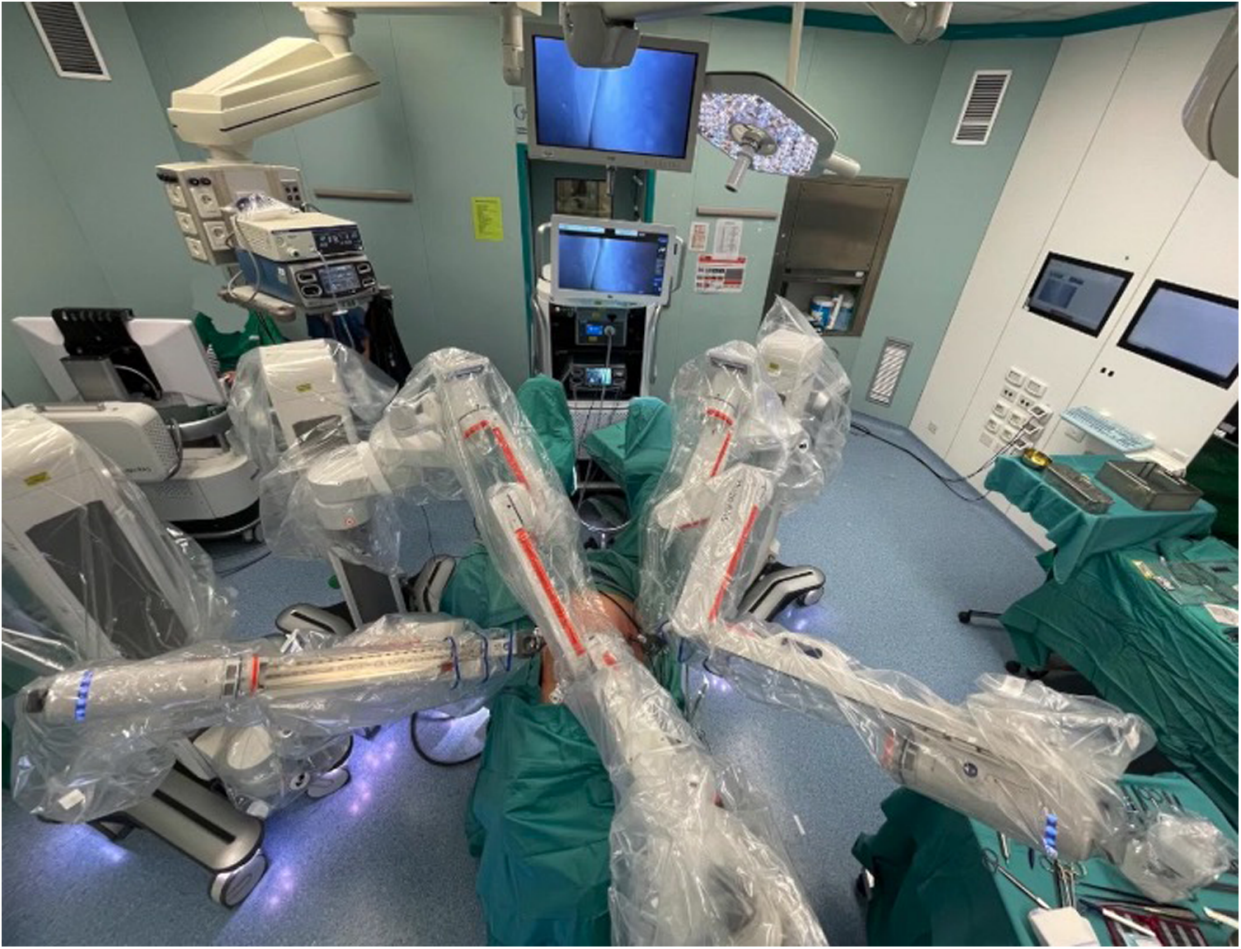
Operating room setting.

Hugo RAS technology seems to be promising, as it includes technical advantages of the systems already in use, adding some potential benefits. Independent arms give free access to the patient from different angles (Figure 3). Furthermore, the open console and eye tracking system allow the surgeon to be aware of his surroundings in the operating room.

**Figure 4:**
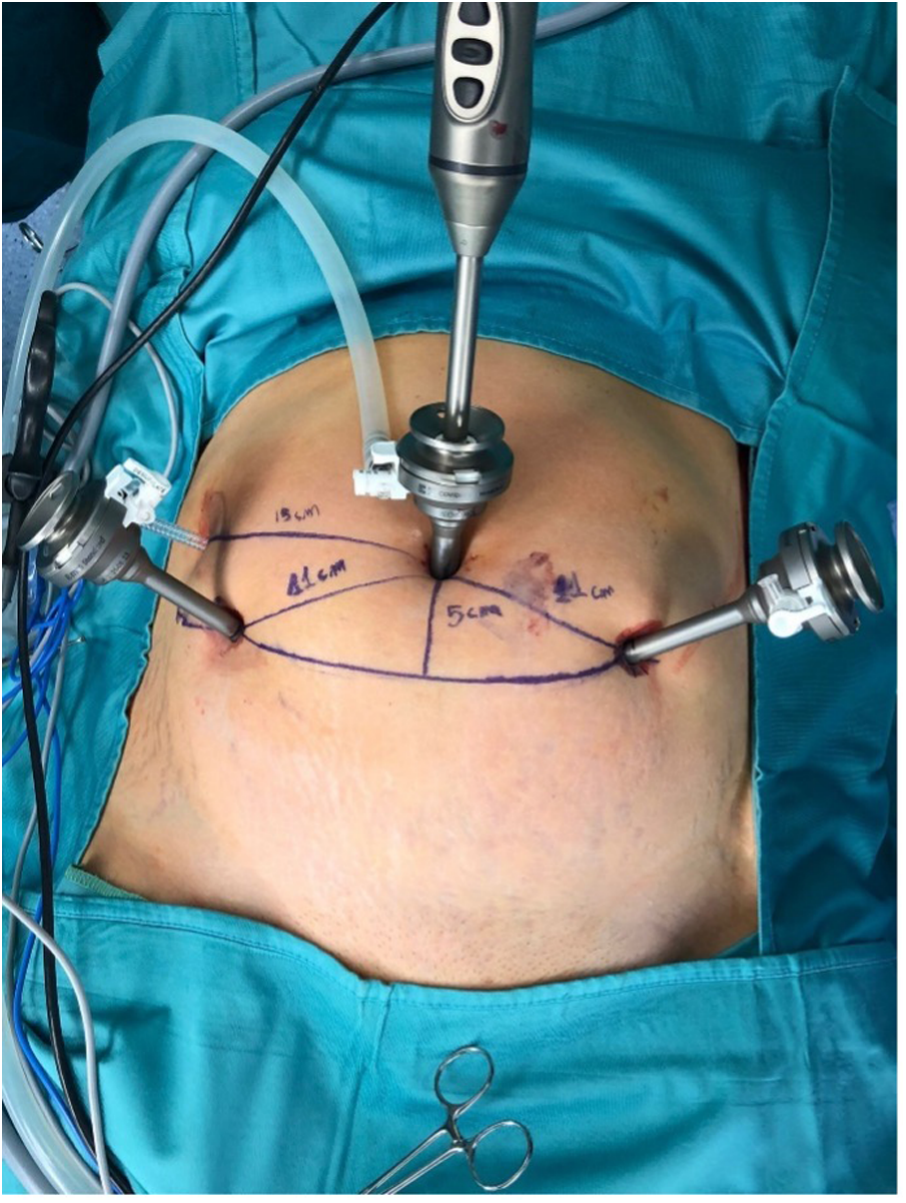
Ports placement.

Trocar positioning is similar to the setting used in a standard laparoscopic sacrocolpopexy, which is relevant since it would give the surgeon the possibility of opting for a convenient and fast conversion to standard laparoscopic setting if in need (Figure 4). Furthermore, the system tower and its visualization system are designed to support both robot-assisted surgery as well as laparoscopy. Besides the advantages in case of conversion, this feature was useful in the 4 cases that required laparoscopic adhesiolysis before docking.

Additionally, the position of the ports is different from standard RSCP setting (where all ports are positioned on the trans-umbilical line), with the two lateral incisions resulting lower on the abdomen. Although all patients were highly satisfied with the overall outcome of the appearance of their scars, this does not offer a clear advantage of this approach over laparoscopic or standard robotic surgery.

The separate arms design allowed a great variety of modifications during docking. Carts position, docking manoeuvres and tilt angles of each arm were adjusted by the first assistant to better match the patient's body type, operation characteristics and surgeon's preferences. In the first few procedures, there were some cases of external clashing of two robotic arms. This did not lead to major time delay or adverse events, thanks to a built-in alarm system that is able to stop the instruments until the operator unblocks them manually. In those rare cases, small adjustments in the docking and tilt angles of the arms were sufficient to provide enough space for each arm extracorporeally.

**Figure 5:**
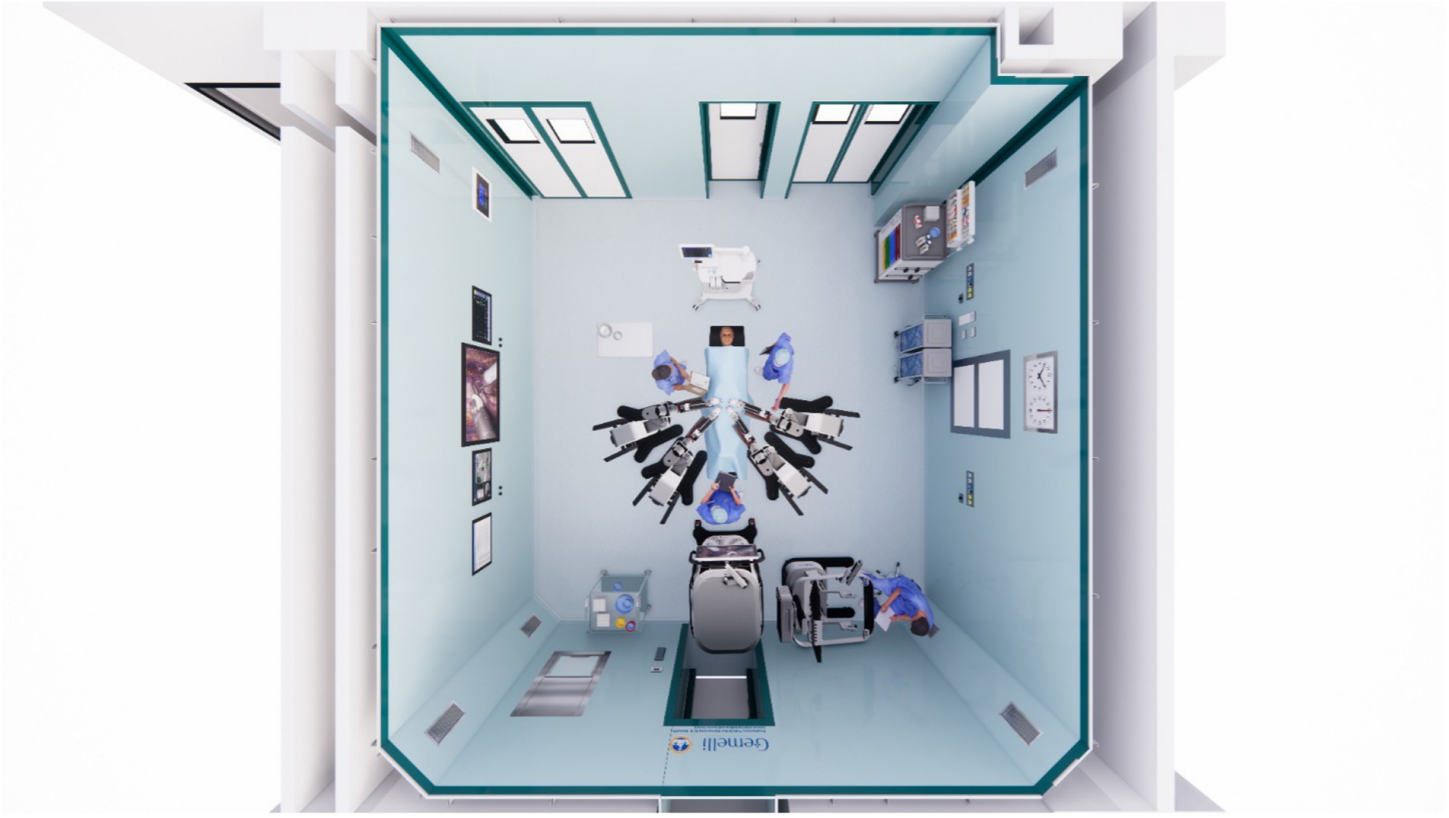
Schematic view of the operating room setting.

The settings provided in Figure 5 may help to minimize the described issue and facilitate access of the assistants to the operating table. Although this was our initial experience, docking times were comparable to previous RSCP performed with the Da Vinci™ platform in our centre, or reported in literature. The separate arms also render the whole system more versatile, allowing it to be moved with greater ease between operating rooms. This might be a great advantage in centres that do not have a dedicated robotic operating room. On the other hand, one of the disadvantages is related to the bigger space occupied by the robotic arms during the procedures (that cover an area of about 3 × 4 m around the operative table) compared to the monolithic DaVinci.

Both surgeons that performed the procedures were satisfied with the open console that enabled them to sit in an upright position and have a direct communication with the surgical team. One of the main disadvantages of this platform is that the docking process for multiple arms might be more time-consuming. However, thanks to an adequate training, our staff in the operating room was able to dock the different arms at the same time, reducing overall time. Another disadvantage is related to the cost effectiveness of this platform compared to the others, that still must be verified in clinical practice.

In a recent report by Raffaelli et al. on the first cases of adrenalectomy performed with this system, an interesting cost-effectiveness analysis was made (16).

According to their analysis, RAS seems to be economically sustainable in a Health Care System where inpatient care reimbursement is based on Diagnosis-Related Groups (DRGs), despite the positive margin (meaning the reimbursement minus the total cost of the operation) being significantly reduced compared to laparoscopy. Furthermore, one of the proposals for reducing costs applicable to robotic platforms, is the implementation of an alternate charging model, where the required instruments for each procedure may be purchased as part of an operation kit to reduce total costs. This may be especially useful in high volume centres that might benefit from “purchasing operations in bulk”.

## Conclusions

This is the first series analyzing RSCP outcomes for POP using the new Hugo RAS system. Our results suggest effectiveness both in objective and subjective outcomes, with minimal intra and post- operative complications, comparable to standard minimally invasive techniques. Large series as well as longer follow-up are without doubt needed to better define advantages and possible disadvantages of this novel system. Our case series may represent the basis of future studies to confirm the safety, efficacy and feasibility of the technique. This was a pilot study, with the idea of pre-analyzing patient data while planning a prospective study with a larger sample.

## Data Availability

The raw data supporting the conclusions of this article will be made available by the authors, without undue reservation.
